# Health-related quality of life and emotional well-being in patients with glioblastoma and their relatives

**DOI:** 10.1007/s11060-020-03614-5

**Published:** 2020-09-09

**Authors:** Pernilla Ståhl, Boglarka Fekete, Ingela Henoch, Anja Smits, Asgeir S. Jakola, Bertil Rydenhag, Anneli Ozanne

**Affiliations:** 1 Department of Neurosurgery, Sahlgrenska University Hospital, Gothenburg, Sweden; 2 Department of Neurology, Sahlgrenska University Hospital, Gothenburg, Sweden; 3 Institute of Health and Care Sciences, Sahlgrenska Academy, University of Gothenburg, Box 457, 40530 Gothenburg, Sweden; 4 Department of Clinical Neuroscience, Institute of Neuroscience and Physiology, Sahlgrenska Academy, University of Gothenburg, Gothenburg, Sweden; 5 Department of Oncology, Sahlgrenska University Hospital, Gothenburg, Sweden; 6 Department of Neuroscience, Neurology, Uppsala University, Uppsala, Sweden

**Keywords:** Astrocytoma grade *ιv*, Glioblastoma, Quality of life, Population-based study, Relatives

## Abstract

**Purpose:**

The health-related quality of life (HRQoL) for patients with glioblastoma is known to be largely affected. Little is known about the HRQoL for relatives and the relationship between these two. To optimize family care, such issues need to be addressed early on, preferably from the time of diagnosis. This study aimed to describe and compare the HRQoL of patients with glioblastoma and their relatives before surgery.

**Methods:**

A prospective cohort study including 89 patients diagnosed with glioblastoma and their relatives. HRQoL (Short Form Health Survey, SF-36) and emotional well-being (hospital anxiety and depression scale, HADS) were analysed with descriptive, comparative and multivariable regression analyses.

**Results:**

Relatives scored worse for mental HRQoL (p < 0.001) and for symptoms of anxiety (p < 0.001) and depression (p = 0.022) compared to patients. The multivariable regression showed an increased risk of affected mental HRQoL in relatives of patients with poor functional status (WHO) (p = 0.01) and higher levels in symptoms of anxiety (p = 0.03), or when relatives had low physical HRQoL themselves (p = 0.01). There was increased risk of affected mental HRQoL in patients with comorbidities (p = 0.003), and when the respective relative showed higher levels in symptoms of anxiety (p = 0.005).

**Conclusion:**

Relatives scored worse for mental HRQoL and emotional well-being than patients, suggesting that HRQoL in patients and relatives might be connected to symptoms of anxiety in the respective individual at disease onset. The results illustrate the need to screen HRQoL and emotional well-being in both patients and relatives from an early stage—before surgery.

## Introduction

Glioblastoma is the most common and aggressive primary malignant brain tumor in adults with an incidence of approximately 3.2/100 000 [1]. The mainstay of treatment for glioblastoma is surgical resection, followed by chemo- and radiotherapy. Despite combined therapy, the median survival for glioblastoma is about 1–2 years [2, 3] and 5-year survival rate at around 5–9% [1, 4, 5].

Patients with glioblastoma present with a variety of symptoms and signs, such as neurological deficits and epileptic seizures, cognitive problems and neuropsychiatric symptoms of anxiety and depression, caused by the tumor itself, tumor-related treatment, or a combination of these factors [6]. These symptoms may all have a negative effect on the health-related quality of life (HRQoL) [7, 8], leading to inactivity [7] and the ability to continue a normal working life [7, 9]. The patient’s accelerating symptoms may also affect the nearest relative’s HRQoL [10]. In studies comparing gender, female generally score lower HRQoL then men [11, 12]. In addition, the individual situation of the patient will be affected by the strains placed upon the family members by the disease and the altered roles within the family—with the relatives themselves being affected by the increased burden imposed upon them [7, 9, 13, 14]. To our knowledge no studies have examined the relationship between the experiences of patients and relatives HRQoL and emotional well-being—before surgery.

To optimize our current de facto palliative oncological care, and develop and implement useful support to promote well-being, information about baseline HRQoL, emotional well-being, and family relationships is necessary. Identifying potential problems at early stage, and addressing these appropriately, is essential and could—in the long run—improve the HRQoL and emotional well-being in this group of patients and their relatives.

It is important to address and fill out this knowledge gap of the relation between patients and relatives before surgery. Relatives are likely to play an essential role in the HRQoL and emotional well-being of the patients with glioblastoma, as well as being affected themselves by the changes caused by the disease. The aim of this study was to describe and compare the HRQoL and emotional well-being in patients with glioblastoma and their relatives before surgery.

## Material and methods

### Participants

**Fig. 1:**
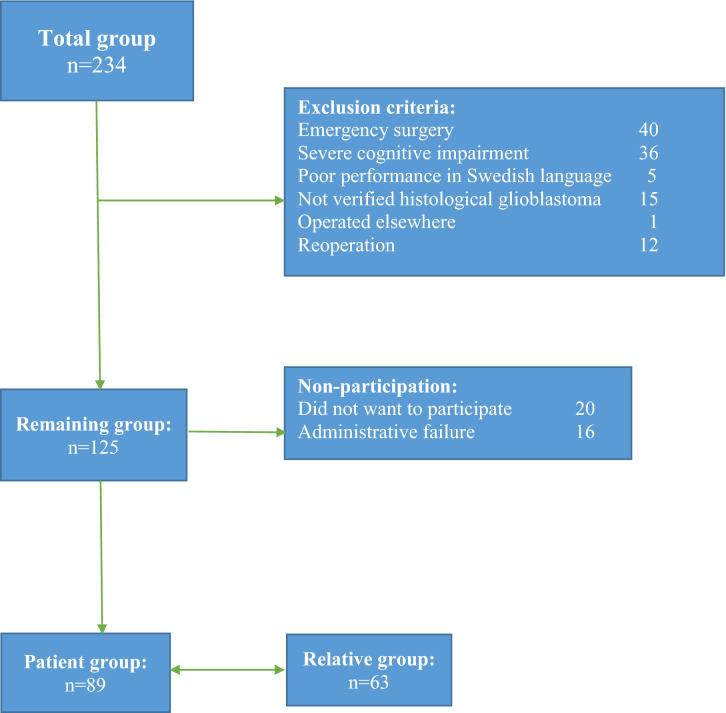
Flow chart showing the sample, exclusions and non-participation

This was a prospective cohort study and patients with glioblastoma were identified from a population-based study at the University Hospital in Gothenburg, Sweden [15]. Patients over 18 years old with a radiological diagnosis of glioblastoma in the South-West region of Sweden with approximately 1.7 million inhabitants (one of the two lager regions in Sweden) were approached prior to surgery. Patients and their relatives were thoroughly informed about the suspected diagnosis. The following exclusion criteria were used: emergency surgery, severe cognitive impairment, poor performance of the Swedish language, operated elsewhere, reoperation. In addition, patients with other histological diagnosis than glioblastoma were excluded after surgery (Fig. 1). For recruitment of relatives, the patients were asked to select a person closest to them (this occurred 1–3 days before surgery), regardless of they were living together or not. The relative was then contacted by the study coordinator and asked to participate in the study.

### Measures

Short Form Health Survey (SF-36) is a validated and reliable questionnaire when measuring HRQoL, both for general population [16, 17] and for patients with brain tumors [18], the questionnaire is generic and one of the most widely used measures of HRQoL in clinical studies. SF-36 includes 36 items, with 35 items divided into eight domains, which are subsequently divided into two major health components—physical component summary (PCS) and mental component summary (MCS) [16, 17]. The domains and dimensions range from 0 to 100 (worst possible health state to best possible health state) [17], with a population mean of 50 and a standard deviation (SD) of 10 [19].

The hospital anxiety and depression scale (HADS) is a validated [20] questionnaire. It consists of a self-assessment scale with 14 items divided into two subscales, including the person’s own experience of anxiety (HADa) and depression (HADd). Items range from 0 to 3 on a four-point Likert scale. The score for each subscale ranges from 0 to 21. Seven points or lower indicate the absence of significant anxiety or depression, scores between 8 and 10 indicate doubtful cases, and scores over 11 indicate definite cases of anxiety or depression [21].

One way to measure functional status is Eastern Cooperative Oncology Group Performance Status (ECOG PS), this questionnaire range from 0 to 5: 0; no limitations of activity, 1; restricted in physically exhausting activity, 2; ambulatory and capable of all self-care, but unable to carry out any work activities, 3; capable of only limited self-care, 4; completely disabled, 5; dead. This was later adapted by the WHO and has the title ECOG/WHO performance status [22, 23].

### Data collection

Data collection occurred from October 2012 to November 2016. Prior to admission for surgery, the patients and relatives received information about the suspected tumor diagnosis. A research nurse distributed the questionnaires to the patients and relatives in connection with enrolment before surgery. The questionnaires were completed by the individual participant without input from others. Patients and relatives scored their own HRQoL and emotional well-being.

The following data were collected from the medical records: gender, age at surgery, days from diagnosis to surgery, first symptom (neurological deficit, epilepsy, cognitive effects, headache, several symptoms, incidental findings), effects of corticosteroids (e.g. relieved headache or improved neurological function), functional status (according to the ECOG/WHO performance status scale), location of the tumor (unilateral, bilateral, central), tumor side (right, left, bilateral), comorbidity (according to an adapted Charlson Comorbidity Index (CCI) [24]) hypertension, diabetes and epilepsy and surgical planning (radical resection, subtotal resection, biopsy).

### Statistics

To present data descriptive statistics were used. Wilcoxon signed-rank test was used for the analysis of paired comparisons between patients and relatives. Mann–Whitney U test was used to analyse differences between groups. Univariable linear regression analysis applying forward selection was performed to test the impact of various variables on SF-36 MCS in patients as well as in relatives. These variables were: age of patient at surgery; days from diagnosis to surgery; first symptom (neurological deficit, epilepsy, cognitive effects); functional status (WHO); location of the tumor; operation side; comorbidity; surgical planning; and the patient’s and relative’s own estimates of SF-36 and HADS. Variables that predicted SF-36 MCS with *p* < 0.20 in the univariable analyses were entered and tested as independent variables in the multivariable stepwise linear regression. All tests were 2-tailed with a significance level of 5%. Statistical analysis was performed using IBM SPSS Statistics 23.

## Results

### Demographic data

A total of 89 patients were included in the study (Fig. 1). Their median age was 64 years, 57 were male and 32 were female. These 89 patients were asked to choose a relative for participating in the study. Of the 63 relatives who participated in the study, 18 were males and 45 were females; 49 were spouses or cohabitants (15 males/34 females), 12 were offspring of adult age (3 males/9 females), one was parent, and one was daughter-in-law. An overview of clinical and demographic data is provided in Table 1. A flow chart with sample numbers, reasons for exclusions and non-participation is shown in Fig. 1. An overview of the estimated self-assessed SF-36 and HADS for the patient group (n = 89), for the patients whose relatives were included (n = 63), and the group of relatives (n = 63) is shown in Table 2. Hypertension was the most common comorbidity, followed by diabetes and epilepsy. There were no differences in terms of age, sex, and days from diagnosis to surgery, comorbidity, or preoperative intention between the patients who participated compared with the drop-outs. When WHO was merged into the groups 0–1 and 2–4 there was a tendency that the WHO was worse against the drop-outs (WHO 2–4 = 33.3% in the drop-outs vs. 15.9% in the participated patients).

**Table 1:** Demographic data for the total group of patients (n 89) as well as for the group of patients whose relatives were included in the study (n 63)

	Patients total group	Patients with relatives
	*n (%)*	*n (%)*
Total sample	89	63
Male/Female	57(64)/32(36)	40(63)/23(37)
	*md (range)*	*md (range)*
Age (in years) at surgery	64 (35–82)	62 (37–77)
Days from diagnosis to surgery	19 (7–72)	20 (7–72)
First symptom		
Neurological deficit	20 (22.5%)	12 (19.0%)
Epilepsy	14 (15.7%)	12 (19.0%)
Cognitive effects	5 (5.6%)	4 (6.3%)
Headache	10 (11.2%)	9 (14.3%)
Several symptoms	36 (40.4%)	23 (36.5%)
Incidental findings	4 (4.5%)	3 (4.8)
Effect of corticosteroids		
Yes	56 (62.9%)	42 (66.7%)
No	3 (3.4%)	1 (1.6%)
Not received medication	3 (3.4%)	2 (3.2%)
Not known	27 (30.3%)	18 (28.6%)
Functional status (WHO)		
0	40 (44.9%)	29 (46.0%)
1	36 (40.4%)	24 (38.1%)
2	9 (10.1%)	6 (9.5%)
3	4 (4.5%)	4 (6.3%)
Localization of tumor		
Unilateral	66 (74.2%)	44 (69.8%)
Bilateral	2 (2.2%)	2 (3.2%)
Central	21 (23.6%)	17 (27.0%)
Operation side		
Right	45 (50.6%)	32 (50.8%)
Left	35 (39.3%)	25 (39.7%)
Bilateral	9 (10.1%)	6 (9.5%)
Comorbidity		
Yes	49 (55.1%)	33 (52.4%)
No	38 (42.7%)	28 (44.4%)
Unknown	2 (2.2%)	2 (3.2%)
Earlier surgery for lower grade of tumor		
Yes	2 (2.2%)	1 (1.6%)
No	87 (97.8%)	62 (98.4%)
Preoperative intention		
Radical	51 (57.3%)	34(54.0%)
Subtotal	29 (32.6%)	23 (36.5%)
Biopsy	9 (10.1%)	6 (9.5%)

Please note that the clinical parameters at baseline did not differ between the two groups

**Table 2:** Overview of reported SF-36 and HADS for all patients (n 89), patients whose relatives were included (n 63), the relatives themselves (n 63), and paired comparisons between patients and their relatives

	Total group of patients n 89	Patients n 63 mean (sd)	Relatives n 63 mean (sd)	Paired comparison between patients (n 63) and relatives (n 63) p-value
SF-36				
PCS	43.6 (11.7)	43.2 (11.0)	57.3 (7.9)	< 0.001*
MCS	37.6 (14.1)	37.2 (13.3)	29.2 (14.7)	< 0.001**
PF	71.0 (30.6)	70.7 (29.9)	91.0 (17.4)	< 0.001*
RP	34.3 (42.7)	26.1 (38.6)	76.6 (35.6)	< 0.001*
BP	69.8 (31.0)	73.6 (29.8)	84.2 (23.7)	0.047*
GH	66.9 (20.7)	66.9 (19.8)	76.7 (17.0)	0.006*
VT	52.0 (27.3)	51.7 (25.7)	50.2 (22.9)	0.813
SF	62.2 (30.0)	58.9 (27.7)	60.1 (27.1)	0.706
RE	44.7 (45.2)	41.9 (45.5)	39.2 (43.3)	0.774
MH	61.9 (23.6)	63.0 (22.8)	52.6 (20.6)	0.001**
HADS				
Anxiety mean (%)	7.0 (4.9)	6.8 (4.7)	9.4 (4.7)	< 0.001**
Probable cases n (%)	19 (21.3)	13 (21.3)	23 (37.7)	
Possible cases n (%)	13 (14.6)	10 (16.4)	17 (27.9)	
Non-cases n (%)	54 (60.7)	38 (62.3)	21 (34.4)	
Depression mean (%)	5.6 (5.1)	5.4 (4.8)	6.7 (4.8)	0.022**
Probable cases n (%)	16 (18.0)	11 (18.0)	16 (26.2)	
Possible cases n (%)	11 (12.4)	5 (8.2)	7 (11.5)	
Non-cases n (%)	60 (67.4)	45 (73.8)	38 (62.3)	

The eight domains in SF-36 are Physical Functioning (PF), Role Physical (RP), Bodily Pain (BP), General Health (GH), Vitality (VT), Social Functioning (SF), Role Emotional (RE), and Mental Health (MH)—and the two major dimensions of health are Physical Component Summary (PCS) and Mental Component Summary (MCS)

*Patients scored worse than relatives

**Relatives scored worse than patients

### Comparisons between patients and relatives

Paired comparisons between patients and relatives showed that patients scored worse levels for the physical parameters in SF-36; physical functioning (p < 0.01), role physical (p < 0.01), bodily pain (p = 0.047), general health (p = 0.007), and total PCS (p < 0.001), while relatives scored lower levels for the psychologic parameters in SF-36, i.e., mental health (p = 0.001) and total MCS (p < 0.001). In addition, relatives scored significantly worse symptoms for HADa (p < 0.001) and HADd (p = 0.022) (Table 2).

Compared to male relatives, female relatives scored worse levels in SF-36 vitality (p = 0.018), SF (p = 0.037), and in HADd (p = 0.016). In the case of patients, no differences were found between males and females.

### The relationship between patients’ and relatives’ mental HRQoL and independent variables

As stated, all variables with p-values < 0.20 in the univariable analysis were entered in a step-wise multivariable regression model (Table 3). Of these, the presence of comorbidity (p = 0.003) in patients and HADa (p = 0.005) in relatives retained their significance in the multivariable model, i.e. identified as factors that affected patients’ mental health (Table 3).

**Table 3:** Overview of the univariable and multivariable analyses in the linear regression model in the group of patients (n = 62)

	Patients SF-36 MCS					
	Univariable		Multivariable		Collinearity	
Variables	Beta (95% CI)	p-value	Beta (95% CI)	p-value	Tolerance	VIF
Constant						
Patient data						
Age at surgery	0.078 (− 0.250, 0.467)	0.55				
Days from diagnosis to surgery	− 0.097 (− 0.393, 0.177)	0.45				
Effect of dexamethasone	− 0.227 (− 10.078, 0.524)	0.08				
Neurological deficit as first symptom	0.198 (− 1.833, 15.064)	0.12				
Epilepsy as first symptom	− 0.013 (− 9.060, 8.178)	0.92				
Cognitive effects as first symptom	0.063 (− 17.239, 10.428)	0.62				
Functional status (WHO)	− 0.172 (− 6.432, 1.244)	0.18				
Localization of tumor	0.032 (− 3.349, 4.3)	0.80				
Operation side	− 0.019 (− 5.543, 4.791)	0.89				
Comorbidity	− 0.347 (− 14.054, − 2.489)	0.006	− 0.359 (− 14.029, − 3.131)	0.003	0.999	1.001
Surgical planning	− 0.185 (− 8.731, 1.366)	0.15				
SF-36 PCS	0.015 (− 0.294, 0.331)	0.91				
Data on relatives						
Gender	− 0.099 (− 10.353, 4.577)	0.44				
SF-36 PCS	− 0.035 (− 0.494, 0.377)	0.79				
SF-36 MCS	0.283 (0.032, 0.481)	0.03				
HADa	0.323 (− 1.612, − 0.224)	0.01	− 0.337 (− 1.605, − 0.309)	0.005	0.999	1.001
HADd	− 0.212 (− 1.526, 0.133)	0.10				

Regarding the group of relatives, variables with p-values < 0.20 also were entered in a step-wise multivariable regression model (Table 4). Of these, the presence of patients’ functional status (WHO) (p = 0.01) and HADa (p = 0.03), as well as SF-36 PCS (p = 0.01) in relatives retained their significance in the multivariable model, i.e. those were factors that affected relatives mental health (Table 4).

**Table 4:** Overview of the univariable and multivariable analyses in the linear regression model in the group of relatives (n = 61)

	Relatives SF-36 MCS					
	Univariable		Multivariable		Collinearity	
Variables	Beta (95% CI)	p-value	Beta (95% CI)	p-value	Tolerance	VIF
Constant						
Patient data						
Gender	0.136 (− 3.537, 11.689)	0.29				
Age at surgery	0.171 (− 0.125, 0.651)	0.18				
Days from diagnosis to surgery	− 0.115 (− 0.452, 0.170)	0.37				
Neurological deficit as first symptom	0.067 (− 6.936, 11.864)	0.60				
Epilepsy as first symptom	− 0.104 (− 13.193, 5.547)	0.42				
Cognitive effects as first symptom	− 0.226 (− 28.174, 1.383)	0.08				
Functional status (WHO)	− 0.367 (− 10.079, − 2.147)	0.003	− 0.420 (− 10.454, − 3.228)	0.01	0.984	1.016
Localization of tumor	− 0.005 (− 4.265, 4.088)	0.97				
Operation side	0.053 (− 4.444, 6.786)	0.68				
Comorbidity	− 0.014 (− 7.047, 6.330)	0.92				
Surgical planning	− 0.088 (− 7.488, 3.656)	0.49				
SF-36 PCS	0.187 (− 0.088, 0.589)	0.15				
SF-36 MCS	0.283 (0.039, 0.586)	0.03				
HADa	− 0.267 (− 1.582, − 0.050)	0.04	− 0.248 (− 1.439, − 0.076)	0.03	0.97	1.031
HADd	− 0.289 (− 1.620, − 0.118)	0.02				
Data on relatives						
Gender	− 0.208 (− 14.671, 1.348)	0.10				
SF-36 PCS	− 0.305 (− 1.017, − 0.113)	0.02	− 0.307 (− 0.972, − 0.153)	0.01	0.963	1.038

## Discussion

In this study the mental HRQoL of patients with glioblastoma and their relatives were found to be connected to symptoms of anxiety in the respective individual. In addition, there were differences between the patients and relatives in terms of self-reported HRQoL and emotional well-being. Relatives scored worse for items covering mental HRQoL and reported more frequent symptoms of anxiety and depression than patients.

It is well known that HRQoL and emotional well-being deteriorate in patients with glioblastoma who have had—or are about to undergo—tumor treatment [7, 25, 26]. Anxiety and emotional wellbeing may be more skewed towards patients with major neurological deficits such as significant aphasia or a dominant side hemiparesis. However, this has not been analysed in the study. In this study, relatives reported worse mental states than patients, including worse scores for MCS, impaired mental health and more symptoms of depression and anxiety. It has previously been shown that relatives are a vulnerable group in terms of mental health, with the relatives’ anxiety being at its highest before chemoradiotherapy and remaining high over time [27]. Worse scores among relatives can be related to a deeper insight into the disease and its prognosis, the personality changes that patients undergo [28, 29], as well as the consequence of these combined factors on family life [27].

Other possible explanations for worse scores in the group of relatives might be uncertainty about the surgery, the fear of being forced to take care of the patient, or being left alone. These feelings can at the other hand also be mixed with feelings of gratitude and privilege of taking care of the patient [29, 30].

There was a relation between patients and relatives with regard to symptoms of anxiety and MCS. This indicates that if impaired mental HRQoL and/or anxiety are present in one group, there is a likelihood that the other group will experience anxiety and/or lower mental HRQoL. The results help to identify especially vulnerable persons, such as patients with comorbidities and patients that have relatives with symptoms of anxiety.

Little is known about how relatives and patients affect each other mentally, especially in case of a glioblastoma diagnosis. Although the notion of close relatives influencing each other seems intuitively an obvious, there are few data on this subject. A previous study did identify a relationship between male relatives and patients with cancer regarding their mental HRQoL. In the same study, however, no relationship was found between female relatives and patients with cancer in terms of mental HRQoL [31].

In the present study, female relatives scored worse levels than male relatives for vitality and social functioning in SF-36, and for symptoms of depression in HAD. Little is known about gender-related differences occurring with relatives of patients with glioblastoma, but it is known that females generally score lower HRQoL than men [11, 12], and it is therefore relevant to study. A previous study reported similar findings—with female relatives of patients with cancer scoring worse overall HRQoL than male relatives [31]. Regardless of whether or not gender is a contributing cause of the reported differences in HRQoL, our data show that relatives estimate themselves worse than patients. Compared with a normative database with selected age and gender-matched sample [19] patients included in our study have lower estimates in HRQoL. The fact that both patients and relatives have affected HRQoL already before surgery illustrates the importance of a person-centered support already early in health care.

Higher age can be an indirect factor that negatively affects HRQoL and emotional well-being. Although some studies indicate that elderly patients still benefit from treatment [32, 33], increasing age will increase the probability of comorbidity, which has a negative impact on mental HRQoL for patients. Thus, the present study has identified comorbidity as a factor contributing towards patients deteriorated mental HRQoL. However, since hypertension was the most common comorbidity it is not possible to ratiocinate too much. Likewise, at older age, lower physical HRQoL can be presumed in patients with glioblastoma and this can affect patients mental HRQoL.

As expected, patients scored lower physical HRQoL than their relatives. Physical impairment can be one of the symptoms of glioblastoma and decreased physical function in patients can affect relatives negatively. Relatives to patients with low functional status (WHO) had a higher risk of poor mental well-being. WHO was still a significant factor despite the fact that in the sample group there was a slightly better WHO than in the dropout group.

The relation between patients and their relatives in the present study suggests that a family serves as a unit and needs to be treated as such. Thus, the focus of care should not only be on the ill person, but also include the relative. For this, it will be important for the relatives to feel welcomed, acknowledged and listened to and be considered as an essential part of the family [34]. Furthermore, research needs to be done regarding interventions for relatives of patients with glioblastoma, on which the body of literature regarding brain tumors is small and inconclusive [35].

Therefore, in the care of seriously ill patients, person-centered care is preferred and in case there are close relatives, he or she should be valued as an important part of this care. The typically heavy workload and frenetic pace associated with an acute neurosurgical care unit may make it difficult to provide and maintain this particular type of care, but our data clearly point out the importance of support and attention for relatives already before surgery.

Glioblastoma is incurable and has a poor prognosis and treatment focus more on QoL for the remaining time, not only for the patient but also for the relatives. This study shows that patients’ and relatives’ emotional well-being are dependent on each other and this even before surgery. In the clinical situation, the propose is that patients and relatives are screened for quality of life and mental health before surgery, to design a support model for early interception and support to patients at risk and their respective relatives.

## Conclusion

When a patient is presented with glioblastoma, the disease affects the entire family’s mental health and emotional well-being. This study confirms that HRQoL and emotional well-being are affected in both patients and relatives. In fact, the relatives are more vulnerable at the time point before surgery regarding mental health and emotional well-being than the patients themselves. The results emphasize the importance of providing support, not only for the patients, but also for their relatives, early on at the time point before surgery. It would be attractive if early support, focusing on the caregiver/relative, also benefit patients since close relatives are likely to play an essential role for the wellbeing of patients.

## Strengths and limitations

The strength of this study is the study design with a direct comparison between patients and relatives, allowing a family view that includes awareness of relatives’ well-being. To compare data between patients and relatives, well validated generic questionnaires as SF-36 and HADS were chosen. In future studies, it will be of interest to use disease-specific instruments, which however do not allow comparison of pair’s estimates as was the focus of the present study. From an ethical point of view, the number of instruments should also be minimized on already exposed groups. A further strength is that it focuses on an early time point—prior to surgery—since both patients and relatives are already affected at that point and probably need support other than what is currently provided—and indeed more of it.

Concerning limitations, further knowledge of the dynamics of patients’ and relatives’ HRQoL throughout the entire course of the disease would be highly valuable and follow-up studies will be conducted to this end. A relatively large drop-out, could be a limitation, demonstrated in the flow-chart. Although this is a limitation, it is important to study HRQoL issues in diseases like glioblastoma with short survival, which involve severe and disabling symptoms of cognitive decline, personality changes and neurological deficits—even if this leads to fewer participants and larger drop-out rates. A national population based study would of course be of value, but it was not possible to realize. Further, it would be of value to compare the results with the general population, but updated comparable data were not available. Another limitation is that data on education and cultural background are missing. It can be assumed that there may be different experiences depending on the relationship of the relatives to the patient, but due to small samples, these analyses could not be performed. It should be noted though that patients were asked to choose their closest relative, so even if it was not a partner living together with the patient, it was a person standing close to him/her.

## Data Availability

The datasets generated during and/or analysed during the current study are available from the corresponding author on reasonable request.
